# Long non-coding RNA SOX2OT promotes the stemness phenotype of bladder cancer cells by modulating SOX2

**DOI:** 10.1186/s12943-020-1143-7

**Published:** 2020-02-04

**Authors:** Yonghao Zhan, Zhicong Chen, Shiming He, Yanqing Gong, Anbang He, Yifan Li, Lianghao Zhang, Xuepei Zhang, Dong Fang, Xuesong Li, Liqun Zhou

**Affiliations:** 1 Department of Urology, Peking University First Hospital, The Institute of Urology, Peking University, National Urological Cancer Center, No. 8 Xishiku street, Beijing, 100034 China; 2 Beijing Key Laboratory of Urogenital Diseases (Male) Molecular Diagnosis and Treatment Center, Beijing, 100034 China; 3 Department of Urology, The First Affiliated Hospital of Zhengzhou University, Zhengzhou, 450003 China

**Keywords:** SOX2OT, Cancer stem cell, miR-200c, SOX2, Bladder cancer

## Abstract

**Background:**

Accumulating evidence indicates that long non-coding RNAs (lncRNAs) are potential biomarkers and key regulators of tumour development and progression. SOX2 overlapping transcript (SOX2OT) is a novel lncRNA that acts as a potential biomarker and is involved in the development of cancer and cancer stem cells. However, the clinical significance and molecular mechanism of SOX2OT in bladder cancer are still unknown.

**Methods:**

The expression level of SOX2OT was determined by RT-qPCR in a total of 106 patients with urothelial bladder cancer and in different bladder cancer cell (BCC) lines. Bladder cancer stem cells (BCSCs) were isolated from BCCs using flow cytometry based on the stem cell markers CD44 and ALDH1. Loss-of-function experiments were performed to investigate the biological roles of SOX2OT in the stemness phenotype of BCSCs. Comprehensive transcriptional analysis, RNA FISH, dual-luciferase reporter assays and western blots were performed to explore the molecular mechanisms underlying the functions of SOX2OT.

**Results:**

SOX2OT was highly expressed in bladder cancer, and increased SOX2OT expression was positively correlated with a high histological grade, advanced TNM stage and poor prognosis. Further experiments demonstrated that knockdown of SOX2OT inhibited the stemness phenotype of BCSCs. Moreover, inhibition of SOX2OT delayed xenograft tumour growth and decreased metastases in vivo. Mechanistically, we found that SOX2OT was mainly distributed in the cytoplasm and positively regulated SOX2 expression by sponging miR-200c. Furthermore, SOX2 overexpression reversed the SOX2OT silencing-induced inhibition of the BCSC stemness phenotype.

**Conclusion:**

This study is the first to demonstrate that SOX2OT plays an important regulatory role in BCSCs and that SOX2OT may serve as a potential diagnostic biomarker and therapeutic target in bladder cancer.

## Introduction

Urothelial carcinoma of the bladder (UCB) is the sixth most common malignancy in men and the most common genitourinary malignancy worldwide; its incidence and mortality have significantly increased over the past decade [1–4]. Although clinical treatments, including surgery, radiation therapy, chemotherapy, and immunotherapy, have improved over the past decade, the prognosis of patients diagnosed with BC has not significantly improved [5–7]. The prognosis of patients with bladder cancer is closely related to the stage of their bladder cancer [8, 9]. Treatments become less effective if bladder cancer is diagnosed at advanced stages or with metastasis [10–12]. Therefore, finding promising early detection markers and more efficient and safer therapeutic methods has enormous potential significance for improving the clinical strategies and outcomes of bladder cancer.

Long non-coding RNAs (lncRNAs) are an important group of transcribed RNA molecules that have a length greater than 200 nucleotides [13]. The rapid development of RNA genomics has highlighted the role of lncRNAs in many human diseases, especially in cancers [14–19]. Recent accumulating evidence has indicated that lncRNAs, such as BLACAT2, UCA-1, LNMAT1 and PANDAR, play important regulatory roles in diverse biological processes in bladder cancer [20–25]. SOX2OT (SOX2 overlapping transcript, chromosome 3q26.33) is a novel lncRNA located in the intronic region of the SOX2 gene [26]. Recently, accumulating evidence has indicated that SOX2OT is a powerful biomarker involved in the development of multiple cancers and cancer stem cells (CSCs) [27, 28]. Although SOX2OT has been suggested to act as an oncogene, the underlying mechanism by which SOX2OT-mediated gene expression participates in tumourigenesis remains largely unknown [29, 30]. Recent studies have provided evidence that SOX2OT plays a positive role in the transcriptional regulation of the SOX2 gene, and the dysregulation of SOX2OT expression has been highlighted in multiple cancers and CSCs [31–33]. However, the clinical significance and biological function of SOX2OT in bladder cancer are completely unknown.

In the present study, we showed that SOX2OT expression was significantly upregulated in bladder cancer tissues compared with in the corresponding normal tissues, and its expression was significantly correlated with histological grade, TNM stage and prognosis. Furthermore, knockdown of SOX2OT inhibited the stemness phenotype (self-renewal, migration, invasion and tumourigenicity) of BCSCs by downregulating SOX2 expression. Mechanistically, bioinformatics analysis revealed that SOX2OT expression positively correlated with SOX2 expression, and the RNA fluorescence in situ hybridization (FISH) results revealed that SOX2OT was mainly distributed in the cytoplasm. Further experimental results demonstrated that SOX2OT functioned as a miRNA sponge to positively regulate SOX2 expression by sponging miR-200c in a ceRNA-dependent manner. Furthermore, knockdown of miR-200c reversed the inhibition of SOX2 expression, and SOX2 overexpression reversed the stemness phenotype inhibition of BCSCs induced by silencing SOX2OT. Together, our results suggest that SOX2OT is a powerful tumour biomarker, which highlights its potential clinical utility as a promising therapeutic and diagnostic target of bladder cancer.

## Materials and methods

### Clinical sample collection and cell culture

Fresh bladder cancer tissue samples and pair-matched normal tissue samples were obtained from patients who underwent radical cystectomy. After resection, fresh bladder cancer tissue and pair-matched normal adjacent bladder tissue obtained from the same patient were snap-frozen in liquid nitrogen immediately. Each patient included in this study signed an informed consent form, and this study was approved by Institutional Review Board of Peking University First Hospital Biomedical Research Ethics Committee of Peking University First Hospital, Beijing, China. The normal urothelial cell line SV-HUC-1 and the bladder cancer cell lines SW780, 5637, J82, UM-UC-3, T24, UM-UC-14, HT1367, TUCCUP, RT4, BIU87 and EJ were used in this study. SV-HUC-1, HT1367, T24, J82 and UMUC3 cells were cultured in DMEM (Corning, USA) supplemented with 10% foetal bovine serum (FBS; Biological Industries, USA), while the SW780, 5637, UM-UC-14, TUCCUP, RT4, BIU87 and EJ cells were cultured in RPMI 1640 (Corning, USA) supplemented with 10% FBS (Biological Industries, USA). BCSC-SW780 and BCSC-5637 cells were cultured in DMEM/F-12 supplemented with 20 ng/mL EGF, 20 ng/mL bFGF and 2% B27. The plates were incubated at 37 °C in a humidified 5% CO_2_ atmosphere.

### Flow cytometry analysis assay

BCSCs were isolated from bladder cancer cells (BCCs) using flow cytometry based on the stem cell markers CD44 and ALDH1. The BCSCs were resuspended in DMEM/F-12 supplemented with 20 ng/mL EGF, 20 ng/mL bFGF and 2% B27 and then cultured in RPMI 1640 supplemented with 10% FBS. The cells were incubated at 37 °C in a humidified 5% CO_2_ atmosphere. Cell apoptosis was determined by flow cytometry. Briefly, cells were cultured in normal medium and transfected with the corresponding shRNA. Cells were collected after transfection for 48 h. Cell apoptosis was determined by PE Annexin V apoptosis detection kits (BD Pharmingen, San Diego, CA, USA). Finally, cell apoptosis was determined using flow cytometry (EPICS, XL-4, Beckman, CA, USA). Experiments were repeated at least three times.

### Cell transfection, RNA extraction and quantitative real-time PCR

The plasmid vectors PLKO.1-puro and pLVX-EF1α were purchased from BioVector NTCC, Inc., Guangzhou, China. The microRNA mimic (agomir) and the microRNA inhibitor (antagomir) were purchased from RiboBio, Guangzhou, China. Before transfection, the cells were cultured for 24 h. Then, the cells were transiently transfected with the corresponding vector using Lipofectamine 3000 Transfection Reagent (Invitrogen, Carlsbad, CA, USA) according to the manufacturer’s instructions. After 48 h, cells transfected with the corresponding vector were harvested for quantitative real-time PCR. The stable cell line was established by lenti-virus infection accordingly. Lenti-virus was produced using three vectors system: transfer vector, viral packaging (psPAX2) and viral envelope (pMD2G) at 4:3:1 ratio transfected into 293 T cells. Then, the bladder cancer cells were infected by lentiviruses according to the MOI value (the number of lentiviruses per number of cells). The knockdown and overexpressed stable cell lines were selected with puromycin (2 μg/mL) and blasticidin (10 μg/mL), respectively. Total RNA from the tissues and cells was extracted using TRIzol Reagent (Invitrogen, Carlsbad, CA, USA). The cDNA was reverse transcribed from the total RNA by the PrimeScript RT reagent kit with gDNA Eraser (TaKaRa, Japan). Quantitative real-time PCR was performed using SYBR Premix Ex Taq II (TaKaRa, Japan) and the 7500 Fluorescent Quantitative PCR System (Applied Biosystems Life Technologies, USA), and the results were normalized to β-actin or U6 small nuclear RNA. The detailed primer sequences are listed in Additional file 4: Table S2.

### Western blotting analysis

Total cell lysates were prepared as previously described. Total proteins were separated by 12% SDS–PAGE and transferred to PVDF membranes. The PVDF membranes were blocked with 5% non-fat milk and incubated overnight at 4 °C with the primary antibody anti-SOX2 (1:1000; Abcam, USA) or E-cadherin/N-cadherin/vimentin (1:2000; Cell Signaling Technology, USA). The membranes were then incubated with a secondary antibody (1:5000; Abcam, USA) and visualized with enhanced chemiluminescence using an ECL kit (Beyotime Biotechnology, China).

### Cell proliferation assays

BCSC proliferation was determined by an ethynyl-2-deoxyuridine (EdU) incorporation assay using an EdU Apollo DNA in vitro kit (RiboBio, Guangzhou, China) following the manufacturer’s instructions. For the EdU incorporation assay, 24 h after transfection, the cells were incubated with 100 μl of 50 μM EdU per well for 2 h at 37 °C. Finally, cell fluorescence was visualized using fluorescence microscopy. BCSC proliferation was determined by a colony-formation assay. For the colony-formation assay, BCSCs were seeded in 6-well plates (2 × 10^2^). After incubation for 7 days at 37 °C in a humidified 5% CO_2_ atmosphere, the cells were stained with 0.5% crystal violet and imaged. Finally, their absorbances were determined using a microplate reader (Bio-Rad, USA).

### Cell metastasis assays

The migratory abilities and invasive abilities of BCSCs were determined using wound-healing assays and transwell assays, respectively. For the wound-healing assay, after transfection with the corresponding vector, the cells were incubated for 24 h; then, a wound was created using a sterile 200-μL pipette tip. Finally, cell migration was monitored under an optical microscope (Olympus, Japan), and the migration distance was calculated by HMIAS-2000. For the transwell assay, 5 × 10^4^ cells were seeded into the upper chamber with serum-free medium, and medium with 10% FBS was added into the lower chamber. After incubation for 24 h, the cells remaining in the upper chamber were wiped off, and the cells that had migrated to the bottom surface were fixed with 4% paraformaldehyde and imaged.

### RNA fish

RNA FISH was performed using a fluorescent in situ hybridization kit (RiboBio, China) following the manufacturer’s instructions. The lncRNA SOX2OT FISH probes were also designed and synthesized by the RiboBio Company. Briefly, BCSCs were collected after transfection with the corresponding vector for 48 h and subsequently seeded on glass coverslips. Finally, fluorescence detection was performed with a confocal laser-scanning microscope (Leica, Germany).

### Dual-luciferase reporter assay

Dual-luciferase reporter assays were performed using a Dual-Luciferase Reporter Assay System (Promega, USA) according to the manufacturer’s instructions. Briefly, SOX2OT-WT/MUT and SOX2-WT/MUT were constructed and co-transfected into BSCS-SW780 cells along with agomir-200c/agomir-NC using Lipofectamine 3000 (Invitrogen, USA) and incubated for 48 h. Finally, the luciferase activities were measured using a microplate reader (Bio-Rad, Hercules, CA, USA).

### Tumour sphere formation

The spheroid-formation ability of BCSCs were determined using tumour sphere formation assays and single-cell tumour sphere formation assays, respectively. For the tumour sphere formation assays, BCSCs were collected after transfection with the corresponding vector for 48 h; then, 1 × 10^2^ BCSCs were seeded on an ultra-low attachment surface 24-well plates (Corning, USA). BCSCs were resuspended in DMEM/F-12 supplemented with 20 ng/mL EGF, 20 ng/mL bFGF and 2% B27 and incubated for 7 days at 37 °C. Finally, the spheres were visualized under an optical microscope (Olympus, Japan). For the single-cell tumour sphere formation assays, BCSCs were collected after transfection with the corresponding vector for 48 h; then, one BCSCs were seeded on an ultra-low attachment surface 96-well plates (Corning, USA). BCSCs were resuspended in DMEM/F-12 supplemented with 20 ng/mL EGF, 20 ng/mL bFGF and 2% B27 and incubated for 7 days at 37 °C. Finally, the spheres were visualized under a confocal laser-scanning microscope (Leica, Germany).

### Mouse model experiments

All animal experiments were approved by the Institutional Animal Care and Use Committee (IACUC) of Peking University First Hospital (Beijing, China) and conducted in accordance with its recommendations and ethical regulations. For the tumour xenograft implantation experiment, 1 × 10^5^ SW780 cells were injected subcutaneously into 5-week-old male BALB/c nude mice (Vital River, Beijing, China), which were subsequently sacrificed 8 weeks later. For the metastasis experiment, 1 × 10^5^ 5637-Luc cells were suspended in 200 μL PBS and injected into the lateral tail veins of 5-week-old male B-NDG mice (BIOCYTOGEN, Beijing, China). Four weeks after the injection, mice were anaesthetized with isoflurane (YIPIN Pharmaceutical CO., LTD., Hebei, China). Ten minutes after D-luciferin was injected, sodium salt (150 mg/kg) was injected intraperitoneally, and cancer cells were detected with an in vivo imaging system, Xenogen IVIS (PerkinElmer, MA, USA). The total flux in photons per second was calculated and reported for each mouse’s lung and liver region using Living Image 4.3.1 (PerkinElmer/Caliper).

### Immunohistochemistry and immunofluorescence

For immunohistochemistry, immunostaining was performed on tissue sections collected from nude mice. For immunofluorescence, BCSCs were seeded on glass coverslips after transfection with the corresponding vector for 48 h. Sections were then incubated with primary antibodies against E-cadherin/N-cadherin/vimentin (1:200; Cell Signaling Technology, USA) followed by incubation with the appropriate secondary antibody. Finally, the sections were visualized under an optical microscope (Olympus, Japan) or under a fluorescence microscope (Olympus, Japan). Using computer multimedia technology and fluorescence microscopy, we established a pathology organization analysis and a living cell fluorescence trace system.

### Statistical analyses

All statistical analyses were performed using SPSS version 19.0 software (SPSS, Inc., Chicago, IL, USA). Statistical analyses were performed with Chi-square test, Student’s t-test and one-way ANOVA, as appropriate. Kaplan-Meier survival analysis was used to evaluate the cumulative survival probability. The correlation between SOX2OT expression and SOX2 mRNA expression in BC was examined using Pearson’s correlation analysis. A *p* value of < 0.05 was regarded as statistical difference.

## Results

### SOX2OT expression is upregulated in bladder cancer

**Fig. 1:**
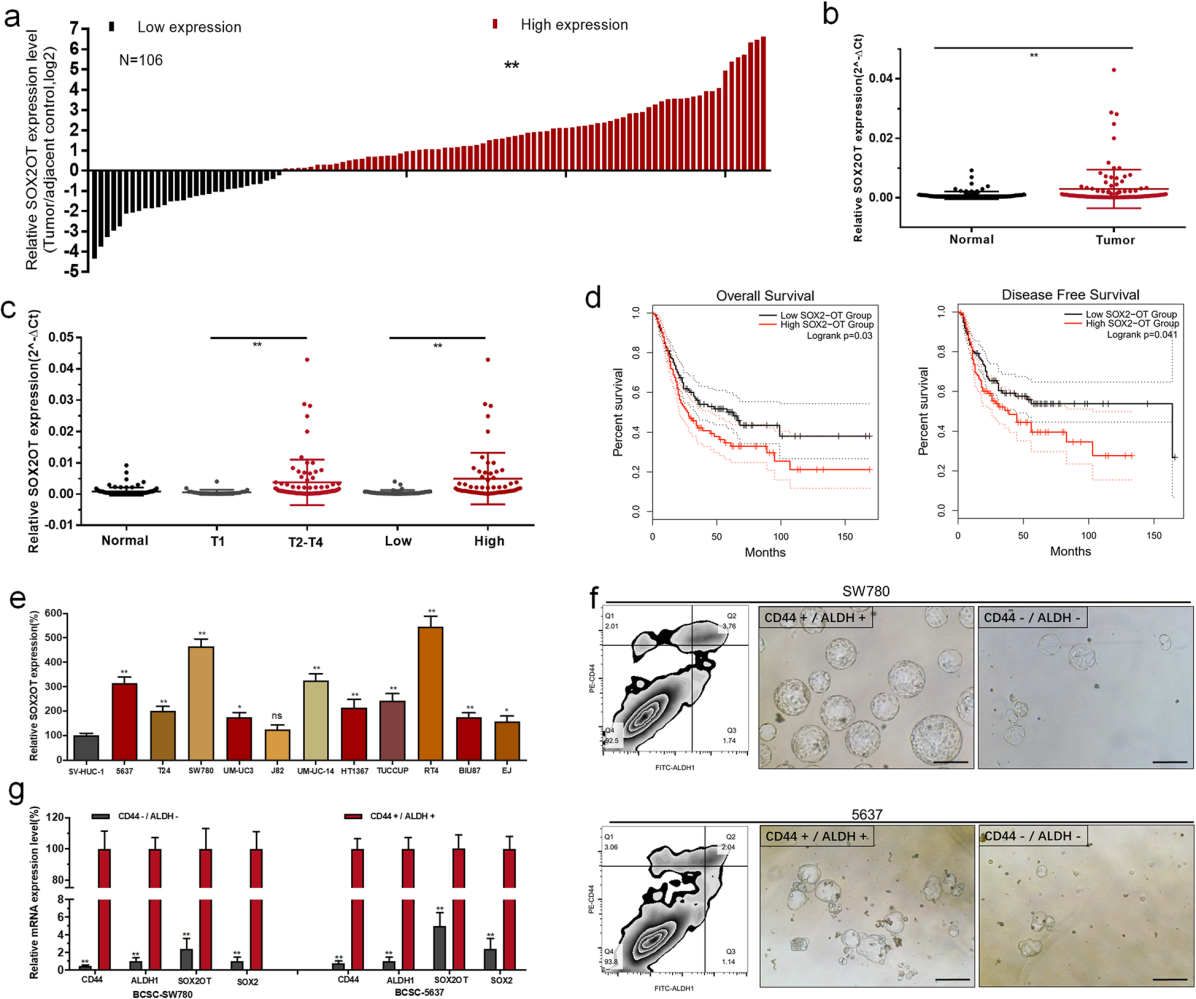
Expression of SOX2OT in bladder cancer. **a** The heights of the columns in the chart represent the log2-transformed fold changes (bladder cancer tissue/normal bladder tissue) in SOX2OT expression in 106 patients with bladder cancer. **b** SOX2OT is upregulated in bladder cancer tissues compared with in the corresponding non-tumour tissues. **c** SOX2OT is upregulated in patients with bladder cancer with an advanced TNM stage and a high histological grade. **d** Higher SOX2OT expression is related to bladder cancer patients’ shorter overall survival (OS) and disease-free survival (DFS) in TCGA-BLCA. **e** SOX2OT is upregulated in bladder cancer cell lines compared with in the normal urothelial cell line. **f** BCSCs were isolated from BCCs using flow cytometry based on the stem cell markers CD44 and ALDH1. **g** SOX2OT and SOX2 expression levels were significantly upregulated in BCSCs compared with in BCNSCs. The data are shown as the mean ± SD. *p < 0.05; **p < 0.01

SOX2OT expression was determined by RT-qPCR in bladder cancer tissues and cell lines. SOX2OT expression was upregulated in 71.7% (76/106) of bladder cancer tissues compared with in the corresponding normal tissue samples (Fig. 1a and b). Moreover, elevated SOX2OT expression was associated with a high histological grade, advanced TNM stage (Fig. 1c) and a poor prognosis (Fig. 1d). SOX2OT expression was upregulated in BC cell lines compared with in the normal urothelial cell line SV-HUC-1 (Fig. 1e). Flow cytometry based on the stem cell markers CD44 and ALDH1 was used to isolate BCSCs from BCCs (Fig. 1f). SOX2OT and SOX2 expression levels were significantly upregulated in BCSCs compared with bladder cancer non-stem cells (BCNSCs) (Fig. 1g). The correlations between SOX2OT expression and the clinical pathological characteristics of patients with urothelial carcinoma of the bladder (UCB) are shown in Table 1. The clinicopathological features of the patients are shown in Additional file 3: Table S1.

**Table 1:** Correlation between SOX2OT expression and clinicopathological features of UCB patients

Parameters Total	Group	Total	SOX2OT expression		*P* value
			High	Low	
Gender	Male	79 (75%)	56 (53%)	23 (22%)	0.751
	Female	27 (25%)	20 (19%)	7 (7%)	
Age (years)	< 60	37 (35%)	26 (25%)	11 (10%)	0.811
	≥ 60	69 (65%)	50 (47%)	19 (18%)	
Tumor size (cm)	< 3 cm	42 (40%)	26 (25%)	16 (15%)	0.071
	≥ 3 cm	64 (60%)	50 (47%)	14 (13%)	
Multiplicity	Single	59 (56%)	38 (36%)	21 (20%)	0.062
	Multiple	47 (44%)	38 (36%)	9 (8%)	
Histological grade	Low grade	48 (45%)	25 (24%)	23 (22%)	0.001 **
	High grade	58 (55%)	51 (48%)	7 (7%)	
Tumor stage (T)	Ta, T1	26 (25%)	12 (11%)	14 (13%)	0.002 **
	T2-T4	80 (75%)	64 (60%)	16 (15%)	
Lymph nodes metastasis	NO	92 (87%)	65 (61%)	27 (25%)	0.768
	YES	14 (13%)	11 (10%)	3 (3%)	

* *P* < 0.05; ** *P* < 0.01; *P* < 0.05 was considered significant

### Knockdown of SOX2OT inhibits BCSC self-renewal

**Fig. 2:**
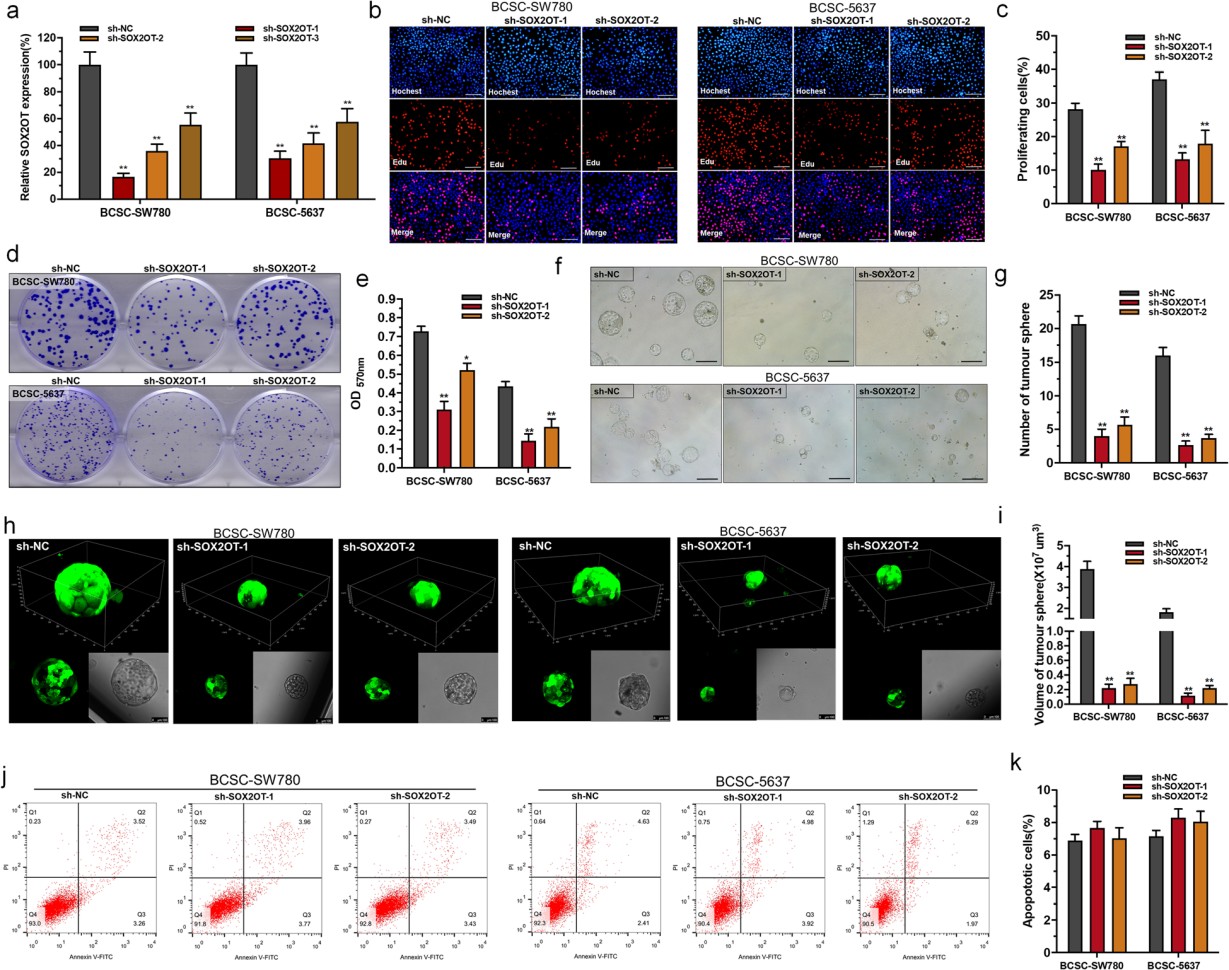
The effect of SOX2OT on the BCSC self-renewal ability and apoptosis. **a**: SOX2OT-specific shRNAs significantly decreased SOX2OT expression in BCSCs. **b** and **c**: Changes in BCSC proliferation were determined using the EdU assay. **d** and **e**: Changes in the colony forming ability of BCSCs were determined using a colony-formation assay. **f** and **g**: Changes in the spheroid-formation ability of BCSCs were determined using tumour sphere formation. **h** and **i**: Changes in the spheroid-formation ability of BCSCs were also determined using single-cell tumour sphere formation. **j** and **k**: There was no difference in apoptosis of BCSCs transfected with the corresponding specific shRNA. The data are shown as the mean ± SD. *p < 0.05; **p < 0.01

We further determined whether SOX2OT regulates the BCSC self-renewal ability. We downregulated SOX2OT expression in BCSCs using SOX2OT-specific shRNAs (Fig. 2a). The cell proliferation of BCSCs were determined using EdU incorporation and colony-formation assays. Cell proliferation inhibition induced by silencing SOX2OT was observed in BCSCs (Fig. 2b-e). Moreover, we found that knockdown of SOX2OT decreased the number and size of tumorspheres (Fig. 2f-i). These results demonstrated that SOX2OT promotes BCSC self-renewal.

### Knockdown of SOX2OT does not affect BCSC apoptosis

We further determined whether SOX2OT regulates BCSC apoptosis. The changes in bladder cell apoptosis were determined using flow cytometry. Regrettably, there was no difference in the apoptosis of BCSCs transfected with the corresponding specific shRNA (Fig. 2j and k). The results indicated that SOX2OT does not affect BCSC apoptosis.

### Knockdown of SOX2OT inhibits cell migration, invasion and epithelial-mesenchymal transition (EMT) of BCSCs

**Fig. 3:**
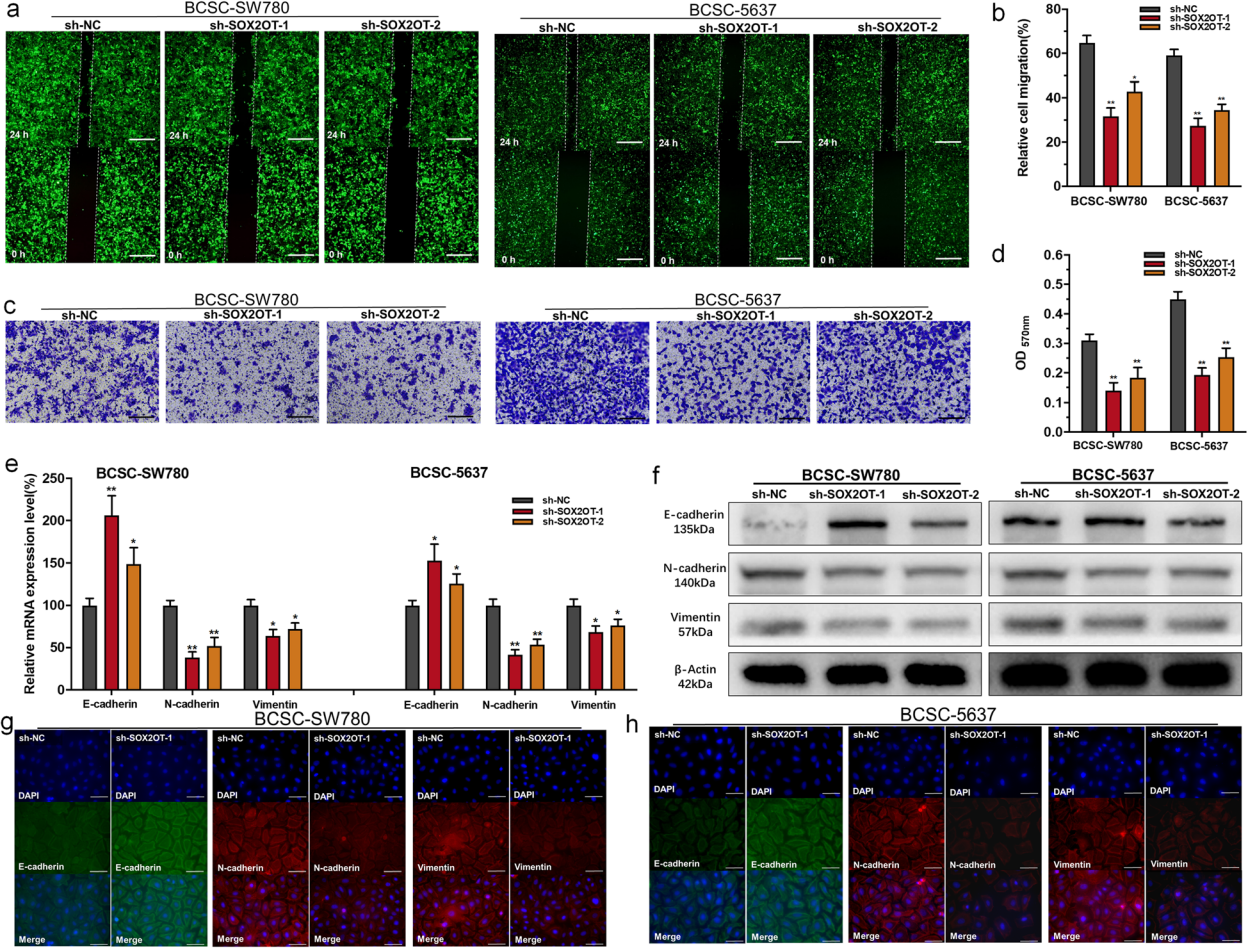
The effect of SOX2OT on BCSC migration, invasion and EMT. **a** and **b**: Changes in the migratory abilities of BCSCs were determined using wound-healing assays. **c** and **d**: Changes in the invasive abilities of BCSCs were determined using transwell assays. **e** and **f**: The expression of EMT markers was determined using RT-qPCR and western blotting. **g** and **h**: The expression of EMT markers was determined using immunofluorescence. Knockdown of SOX2OT increased the expression of E-cadherin and decreased the expression of N-cadherin/vimentin in BCSCs. The data are shown as the mean ± SD. *p < 0.05; **p < 0.01

The migratory abilities of BCSCs were determined using a wound-healing assay. Inhibited cellular migration was observed in BCSCs after silencing SOX2OT (Fig. 3a and b). The invasive abilities of BCSCs were determined using transwell assays. Inhibited cell invasion was observed in BCSCs after silencing SOX2OT (Fig. 3c and d). We further determined whether SOX2OT regulates EMT in BCSCs. The expression levels of EMT markers were determined using RT-qPCR, western blotting and immunofluorescence analyses. Knockdown of SOX2OT increased E-cadherin expression and decreased N-cadherin/vimentin expression in BCSCs (Fig. 3e-h). The results indicated that SOX2OT promotes BCSC migration, invasion and EMT.

### SOX2OT promotes the stemness phenotype of BCSC by modulating SOX2

**Fig. 4:**
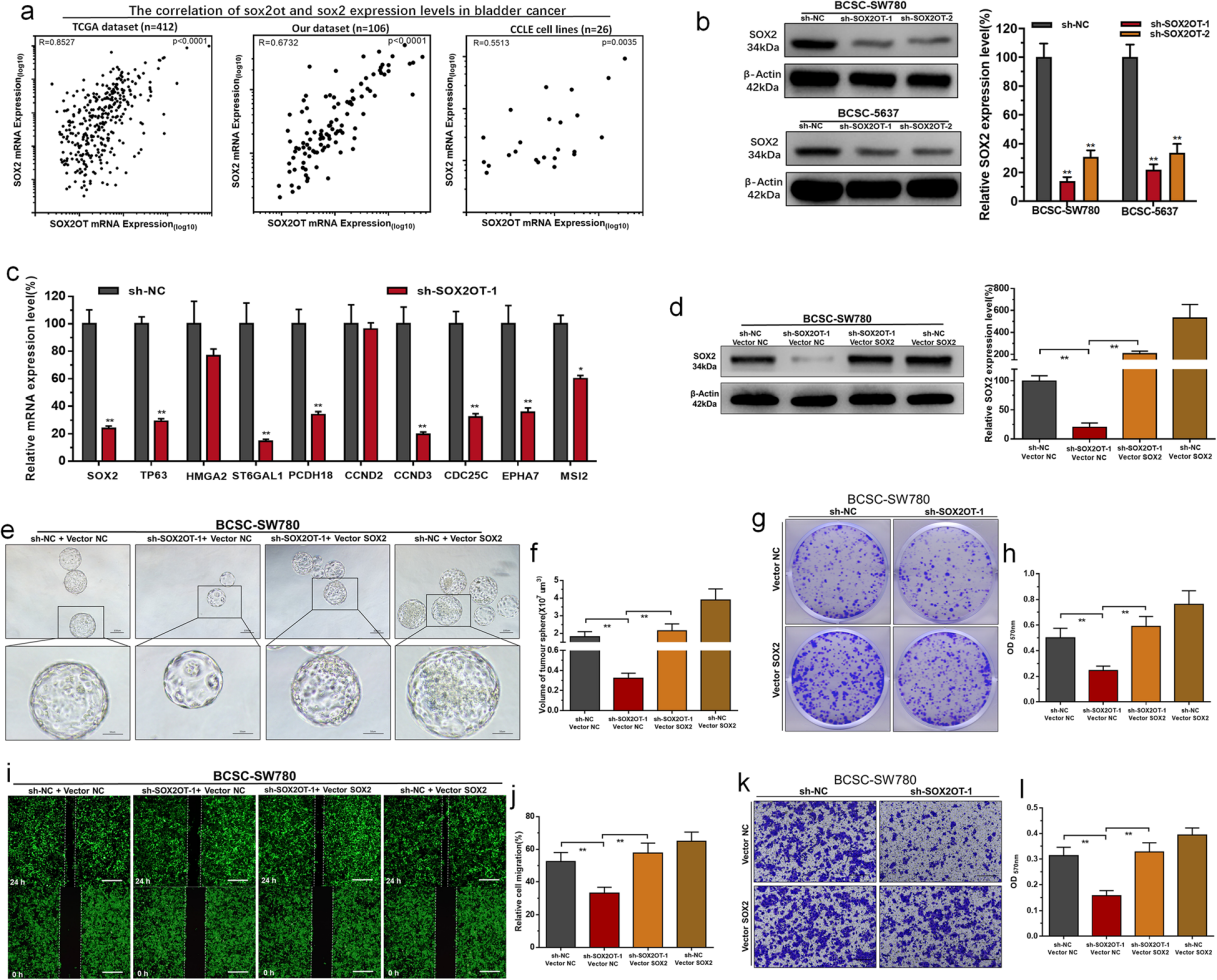
SOX2OT promotes the stemness phenotype of BCSC by modulating SOX2. **a** SOX2OT expression level was positively correlated with SOX2 expression level in BC. **b** Knockdown of SOX2OT decreased SOX2 expression in BCSCs. **c** The expression of SOX2 and SOX2 target genes were determined using RT-qPCR. **d** The SOX2 vector significantly reversed the expression level of SOX2 in BCSCs. **e** and **f** Overexpressing SOX2 significantly reversed the spheroid-formation ability inhibition induced by silencing SOX2OT. **g** and **h** Overexpressing SOX2 significantly reversed the colony forming ability inhibition induced by silencing SOX2OT. **i** and **j** Overexpressing SOX2 significantly reversed the cell migration inhibition induced by silencing SOX2OT. **k** and **l** Overexpressing SOX2 significantly reversed the cell invasion inhibition induced by silencing SOX2OT

To investigate the underlying mechanisms of SOX2OT-mediated biological processes, we performed comprehensive transcriptional analysis by using TCGA and CCLE datasets. The results revealed that SOX2OT expression positively correlated with SOX2 expression in BC, and this correlation was also demonstrated in our tissue samples (Fig. 4a). SOX2 expression was upregulated in our tissue samples (Additional file 1: Figure S1a and S1b), and elevated SOX2 expression was associated with a high histological grade and advanced TNM stage (Additional file 1: Figure S1c). Moreover, Higher SOX2 expression is related to bladder cancer patients’ shorter overall survival (OS) and disease-free survival (DFS) in TCGA-BLCA (Additional file 1: Figure S1d). The correlations between SOX2 expression and the clinical pathological characteristics of UCB patients are shown in Table 2. Furthermore, we found that knockdown of SOX2OT inhibited SOX2 expression (Fig. 4b) and decreased the expression of SOX2 target genes (TP63, ST6GAL1, PCDH18, MSI2, CCND3, CDC25C, and EPHA7) (Fig. 4c). We further determined whether SOX2OT regulates the stemness phenotype of BCSCs in a SOX2-dependent manner. Our results showed that the SOX2-specific vector significantly reversed SOX2 expression in BCSCs (Fig. 4d) transfected with sh-SOX2OT, and SOX2 overexpression significantly reversed the inhibition of BCSC self-renewal (Fig. 4e-h and Additional file 2: Figure S2), migration (Fig. 4i and j) and invasion (Fig. 4k and l) induced by silencing SOX2OT. The results indicated that SOX2OT promotes the stemness phenotype of BCSCs in a SOX2-dependent manner.

**Table 2:** Correlation between SOX2 expression and clinicopathological features of UCB patients

Parameters Total	Group	Total	SOX2 expression		*P* value
			High	Low	
Gender	Male	79 (75%)	59 (56%)	20 (19%)	0.747
	Female	27 (25%)	21 (20%)	6 (6%)	
Age (years)	< 60	37 (35%)	25 (24%)	12 (11%)	0.166
	≥ 60	69 (65%)	55 (52%)	14 (13%)	
Tumor size (cm)	< 3 cm	42 (40%)	27 (25%)	15 (14%)	0.030 *
	≥ 3 cm	64 (60%)	53 (50%)	11 (10%)	
Multiplicity	Single	59 (56%)	42 (40%)	17 (16%)	0.251
	Multiple	47 (44%)	38 (36%)	9 (8%)	
Histological grade	Low grade	48 (45%)	30 (28%)	18 (17%)	0.005 **
	High grade	58 (55%)	50 (47%)	8 (8%)	
Tumor stage (T)	Ta, T1	26 (25%)	16 (15%)	10 (9%)	0.057
	T2-T4	80 (75%)	64 (60%)	16 (15%)	
Lymph nodes metastasis	NO	92 (87%)	69 (65%)	23 (22%)	0.772
	YES	14 (13%)	11 (10%)	3 (3%)	

* *P* < 0.05; ** *P* < 0.01; *P* < 0.05 was considered significant

### SOX2OT positively regulates SOX2 expression by sponging miR-200c

**Fig. 5:**
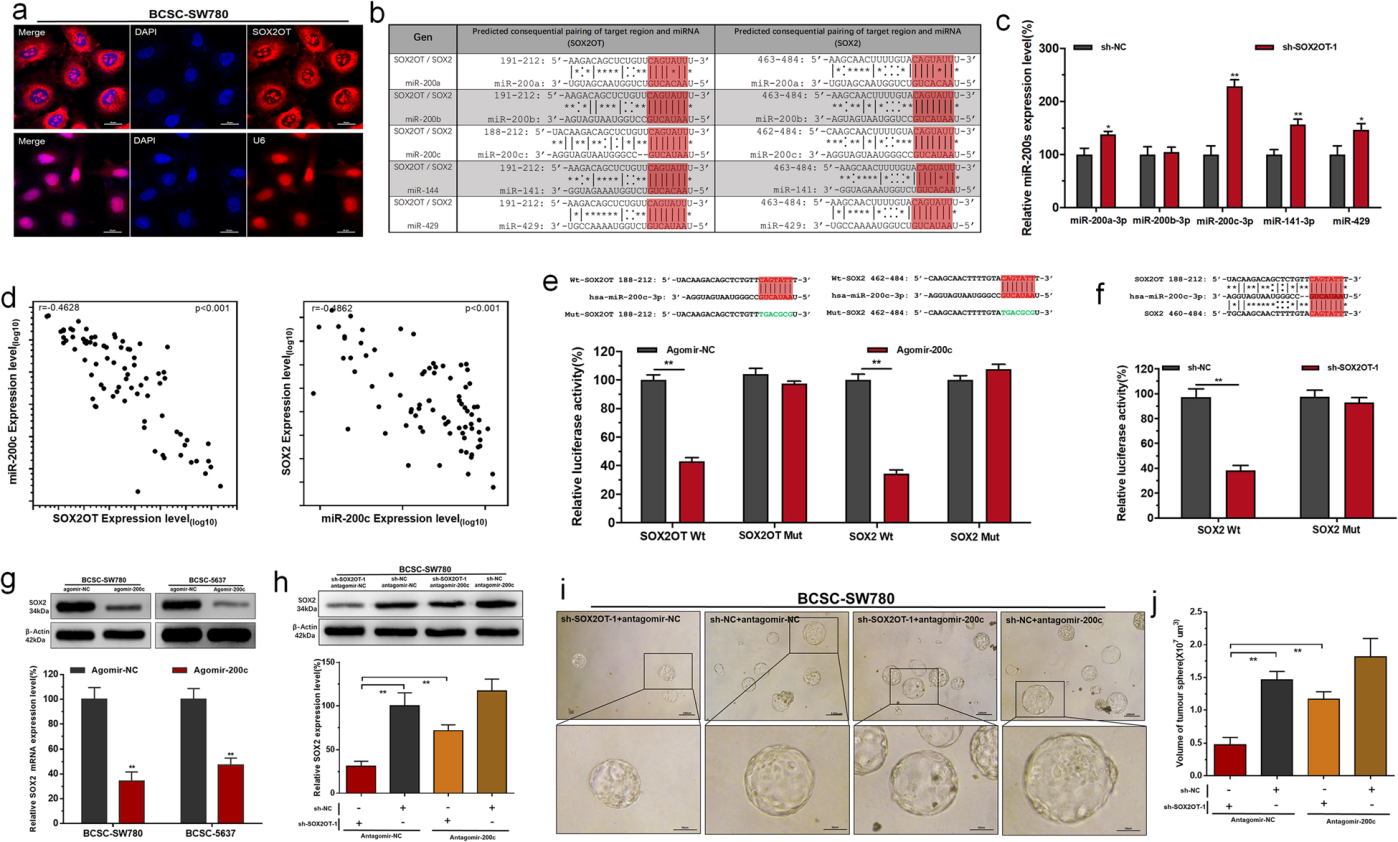
SOX2OT positively regulates SOX2 expression by sponging miR-200c. **a** The RNA FISH results revealed that SOX2OT was distributed mostly in the cytoplasm of BCSCs. **b** SOX2OT and SOX2 have common putative binding sites within the miR-200 cluster. **c**: Knockdown of SOX2OT increased miR-200 s expression in BCSCs. **d** miR-200c expression negatively correlated with SOX2OT/SOX2 expression in bladder cancer tissues. **e** SOX2OT/SOX2-Wt and Agomir200c co-transfection significantly inhibited luciferase activity, and SOX2OT/SOX2-Mut and Agomir200c co-transfection failed to affect luciferase activity. **f** Knockdown of SOX2OT decreased the luciferase activity of cells transfected with SOX2-Wt. **g** Overexpressing miR-200c decreased SOX2 expression in BCSCs. **h**: Knockdown of miR-200c increased SOX2 expression in BCSCs transfected with sh-SOX2OT. **i** and **j** Knockdown of miR-200c significantly reversed the spheroid-formation ability inhibition induced by silencing SOX2OT.The data are shown as the mean ± SD. *p < 0.05; **p < 0.01

The subcellular localization of lncRNAs is closely related to their biological function and potential molecular roles. First, we detected the subcellular localization of SOX2OT using RNA FISH. The RNA FISH results showed that SOX2OT was distributed mostly in the BCSC cytoplasm (Fig. 5a). Through searching in online bioinformatics database, bio-information analysis predicted that SOX2OT and SOX2 have common putative binding sites within the miR-200 cluster (Fig. 5b). The detailed microRNA prediction results are shown in Additional file 5: Table S3. Then, we found that knockdown of SOX2OT increased the expression level of miR-200c in BCSCs (Fig. 5c), and miR-200c expression negatively correlated with SOX2OT and SOX2 expression in BC (Fig. 5d). The results of dual-luciferase reporter assay showed that the co-transfection of SOX2OT/SOX2-Wt with Agomir200c significantly inhibited the luciferase activity, but the co-transfection of SOX2OT/SOX2-Mut with Agomir200c failed to affect the luciferase activity (Fig. 5e). Furthermore, knockdown of SOX2OT decreased the luciferase activity of cells transfected with SOX2-Wt (Fig. 5f). We further determined whether SOX2OT regulates SOX2 expression in BCSCs in a miR-200-dependent manner. We found that miR-200c overexpression inhibited SOX2 expression in BCSCs (Fig. 5g). Moreover, knockdown of miR-200c reversed the SOX2 expression inhibition of BCSCs induced by silencing SOX2OT (Fig. 5h). Moreover, knockdown of miR-200c significantly reversed the spheroid-formation ability inhibition induced by silencing SOX2OT (Fig. 5i and j). These results indicated that SOX2OT positively regulates SOX2 expression by sponging miR-200c in BCSCs.

### Knockdown of SOX2OT inhibits BCSC growth and tumourigenicity in vivo

**Fig. 6:**
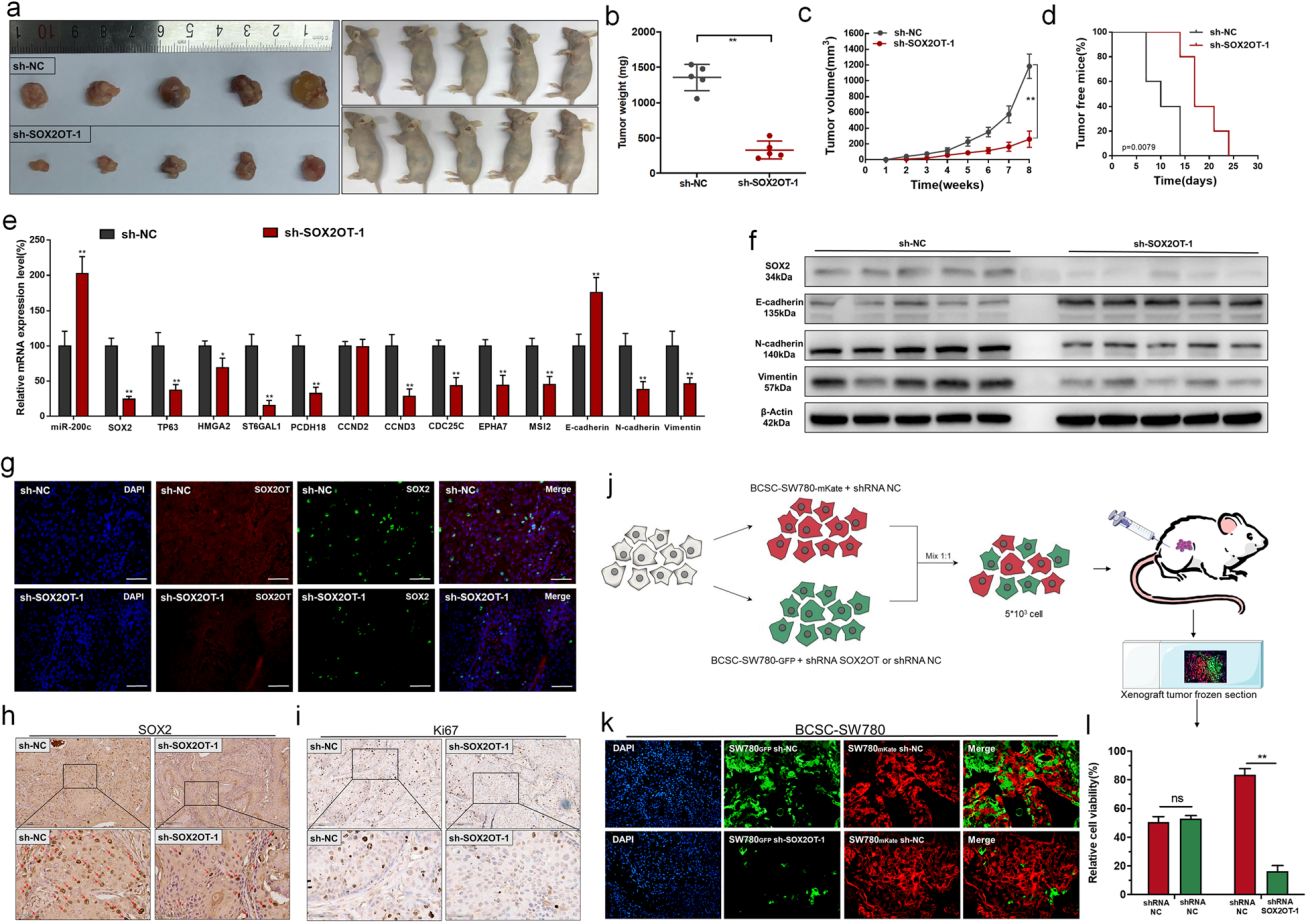
The effect of SOX2OT on BCSC growth and tumourigenicity in vivo. **a** Tumours collected from mice are shown. **b** Tumour weights of the sh-SOX2OT and sh-NC treatment groups were measured and analysed. **c** Tumour volume curves of the sh-SOX2OT and sh-NC treatment groups were measured and analysed. **d** The tumour-free proportions of the sh-SOX2OT and sh-NC treatment groups were measured and analysed. Knockdown of SOX2OT inhibited bladder cancer cell growth in vivo. **e** and **f** Knockdown of SOX2OT increased miR-200c expression, decreased SOX2 and SOX2 target gens expression, and inhibited EMT in BCCs in vivo. **g**: Knockdown of SOX2OT decreased SOX2 expression, and SOX2OT and SOX2 were colocalized in BCCs in vivo. **h** and **i** Knockdown of SOX2OT decreased SOX2 and ki67 expression in BCCs in vivo. **j** Schematic diagram of the cell fluorescence trace system. **k** and **l** Knockdown of SOX2OT significantly decreased the proportion of sh-SOX2OT cells in xenografts. The data are shown as the mean ± SD. *p < 0.05; **p < 0.01

The cell growth of different treatment groups in vivo was determined using xenograft generation. Tumours collected from mice were exhibited and measured (Fig. 6a). The tumour weight of the sh-NC treatment group was greater than that of the sh-SOX2OT group (Fig. 6b). Tumour growth in the sh-NC treatment group was faster than that in the sh-SOX2OT group (Fig. 6c). The proportion of tumour-free mice in the sh-SOX2OT group was higher than that in the sh-NC treatment group (Fig. 6d). We found that knockdown of SOX2OT inhibited SOX2 expression and downregulated the expression of SOX2 target genes (TP63, ST6GAL1, PCDH18, MSI2, CCND3, CDC25C, and EPHA7) in vivo (Fig. 6e). Furthermore, knockdown of SOX2OT inhibited BCC EMT in vivo (Fig. 6e and f). Moreover, we found that knockdown of SOX2OT inhibited SOX2 expression (Fig. 6g and h) and ki67 expression (Fig. 6i) in BCCs and SOX2OT and SOX2 were colocalized in BCCs (Fig. 6g) in vivo. The cell tumourigenicity of cells in different treatment groups was also determined using a cell fluorescence trace system in vivo. As shown in Fig. 6j, we determined the effects of SOX2OT on BCSC tumourigenicity using a cell fluorescence trace system. We found that knockdown of SOX2OT significantly decreased the proportion of sh-SOX2OT cells in xenografts (Fig. 6k and l). The results indicated that SOX2OT promotes BCSC growth and tumourigenicity in vivo.

### Knockdown of SOX2OT inhibits bladder cancer metastasis

**Fig. 7:**
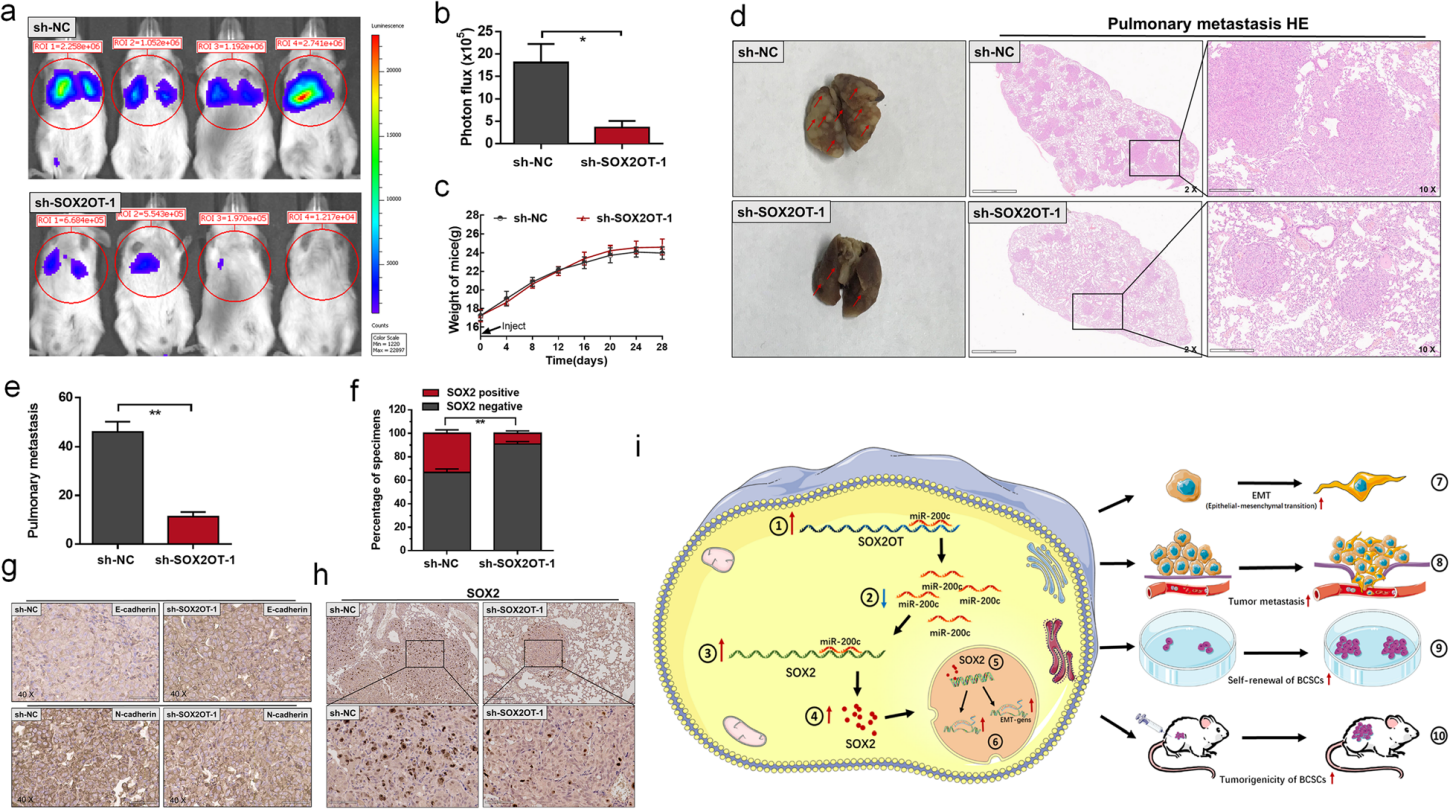
The effect of SOX2OT on bladder cancer pulmonary metastasis and the schematic diagram of the oncogenic role of SOX2OT. **a** and **b** The luciferase signals in the sh-SOX2OT group were remarkably lower than those in the sh-NC group. **c** There was no significant difference between the mouse weight of two treatment group. **d** and **e** The number and size of pulmonary metastases in the sh-SOX2OT group were significantly reduced compared with those in the sh-NC group. **f** and **h** Knockdown of SOX2OT decreased SOX2 expression in pulmonary metastases. **g** The expression of E-cadherin in the sh-SOX2OT group was remarkably higher than that in the sh-NC group. Knockdown of SOX2OT decreased N-cadherin expression in pulmonary metastases. **i** Schematic diagram of the oncogenic role of SOX2OT in bladder cancer

We determined the role of SOX2OT in pulmonary tumour colonization using a whole-body fluorescence imaging system. We found that luciferase signals in the sh-SOX2OT group were remarkably lower than those in the sh-NC group and knockdown of SOX2OT reduced the incidence of bilateral pulmonary metastasis (Fig. 7a and b). There was no significant difference between the mouse weight of two treatment group (Fig. 7c). Haematoxylin-eosin staining was performed on the lung tissue to observe the metastases in the groups. We found that knockdown of SOX2OT significantly reduced the number and size of pulmonary metastases (Fig. 7d and e). Moreover, we found that knockdown of SOX2OT inhibited SOX2 expression (Fig. 6f and h) and inhibited EMT in BCCs in vivo (Fig. 7g). The results indicated that SOX2OT promotes BCSC metastasis and EMT in vivo. As shown in Fig. 7i, SOX2OT functions as a miRNA sponge to positively regulate SOX2 expression by sponging miR-200c and subsequently promotes the stemness phenotype of BCSCs, thus playing an oncogenic role in bladder cancer pathogenesis.

## Discussion

LncRNAs are important members of the non-coding RNA family with transcripts longer than 200 nucleotides [34, 35]. The rapid development of RNA genomics has uncovered that lncRNAs are potential biomarkers and key regulators of stem cell pluripotency and tumourigenesis [20, 36, 37]. Recently, accumulating evidence has shown that lncRNAs can regulate diverse biological processes in multiple ways, such as regulating transcription, sponging miRNA, and modifying epigenetic regulation. For example, CASC9 functions as an oncogene by negatively regulating PDCD4 expression by recruiting EZH2 and subsequently altering H3K27me3 levels in oesophageal squamous cell carcinoma [38]. MRCCAT1 represses NPR3 transcription by recruiting PRC2 to the NPR3 promoter and subsequently activates the p38-MAPK signalling pathway [39]. LNMAT1 epigenetically activates CCL2 expression by recruiting hnRNPL to the CCL2 promoter in bladder cancer [40]. DANCR promotes ROCK1-mediated proliferation and metastasis via crosstalk with miR-335-5p and miR-1972 in osteosarcoma [41].

SOX2OT is a newly identified lncRNA that has been mapped to the human chromosome 3q26.3 locus, and it is involved in the differentiation of embryonic stem cells [42]. Recent studies have provided evidence that SOX2OT plays a key role in transcriptional regulation, and the dysregulation of SOX2OT expression has become highlighted in some somatic cancers. For example, SOX2OT downregulates the expression of SOX3 by regulating miR-194-5p and miR-122, and SOX3 epigenetically activates SOX2OT expression by binding to the SOX2OT promoter and subsequently forming a positive feedback loop in glioblastoma stem cells [33]. Knockdown of SOX2OT in lung cancer decreased EZH2 expression and inhibited cell proliferation by inducing G2/M arrest [32], while knockdown of SOX2OT decreased SOX2 and OCT4 expression and inhibited stem cell pluripotency and tumourigenesis in oesophageal squamous cell carcinoma [43]. Exosomal SOX2OT promotes EMT and stem cell-like properties by regulating SOX2 expression in pancreatic ductal adenocarcinoma [27]. YY1 represses SOX2OT transcription by binding to the SOX2OT promoter and subsequently increases the downregulation of SOX2 expression [28]. Accumulating evidence has indicated that SOX2OT plays a key role in the transcriptional regulation of the SOX2 gene and that SOX2 is a marker for stem-like tumour cells in bladder cancer, suggesting that SOX2OT may play an important regulatory role in BCSCs [44].

In this study, we found that SOX2OT expression was significantly upregulated in bladder cancer tissues compared with in the corresponding normal tissues, and increased SOX2OT expression was positively correlated with an advanced TNM stage, high histological grade and poor prognosis. Moreover, SOX2OT expression was significantly upregulated in BC cell lines compared with in normal urothelial cell lines. Further experiments demonstrated that SOX2OT knockdown inhibited the stemness phenotype of BCSCs. Mechanistically, we found that SOX2OT expression positively correlated with SOX2 expression in bladder cancer, and SOX2OT knockdown inhibited SOX2 expression in BCSCs. Moreover, the RNA FISH results revealed that SOX2OT was distributed mostly in the BCC cytoplasm. The subcellular location of SOX2OT suggests that SOX2OT may function as a ceRNA to regulate the expression of SOX2-related miRNA, and bioinformatics analysis predicted that SOX2OT and SOX2 have common putative binding sites within the miR-200 cluster. Further experimental results demonstrated that SOX2OT functions as a miRNA sponge to positively regulate SOX2 expression by sponging miR-200c and subsequently promoting the stemness phenotype of BCSCs. Moreover, we found that SOX2OT promotes the stemness phenotype of BCSCs in a SOX2-dependent manner and SOX2OT regulates SOX2 expression in BCSCs in a miR-200c-dependent manner.

## Conclusions

Our study revealed that SOX2OT functions as a miRNA sponge to positively regulate the expression of SOX2 by sponging miR-200c and subsequently promoting the stemness phenotype of BCSCs, thus playing an oncogenic role in bladder cancer pathogenesis. The results of this study provide a new basis for studying the mechanism of the occurrence and development of bladder cancer. Cumulatively, our results suggest that SOX2OT is a powerful tumour biomarker, which highlights its potential clinical utility as a promising diagnostic and therapeutic target in bladder cancer.

## Supplementary information

**Additional file 1: Figure S1.** Expression of SOX2 in bladder cancer. a: The heights of the columns in the chart represent the log2-transformed fold changes (bladder cancer tissue/normal bladder tissue) in SOX2 expression in 106 patients with bladder cancer. b: SOX2 is upregulated in bladder cancer tissues compared with in the corresponding non-tumour tissues. c: SOX2 is upregulated in patients with bladder cancer with an advanced TNM stage and a high histological grade. d: Higher SOX2 expression is related to bladder cancer patients’ shorter overall survival (OS) and disease-free survival (DFS) in TCGA-BLCA.

**Additional file 2: Figure S2.** Overexpressing SOX2 significantly reversed BCSC proliferation inhibition induced by silencing SOX2OT. a and b: Overexpressing SOX2 significantly reversed BCSC proliferation inhibition induced by silencing SOX2OT.

**Additional file 3: Table S1.** Summary of clinicopathological features of tissues of bladder cancer.

**Additional file 4: Table S2.** The primer sequences included in this study.

**Additional file 5: Table S3.** Results of Bioinformation analysis.

## Data Availability

The dataset(s) supporting the findings of this study are included within the article.

## Abbreviations

ALDH1: Aldehyde dehydrogenase 1
BCCs: Bladder cancer cells
BCNSCs: Bladder cancer non-stem cells
BCSCs: Bladder cancer stem cells
CCLE: Cancer Cell Line Encyclopedia
ceRNA: Competing endogenous RNA
EdU: Ethynyl-2-deoxyuridine
EMT: Epithelial-mesenchymal transition
FISH: Fluorescence in situ hybridization
LncRNA: Long non-coding RNA
OS: Overall survival
qRT-PCR: Quantitative real-time PCR
SOX2: SRY-box 2
SOX2OT: SOX2 overlapping transcript
TCGA: The Cancer Genome Atlas

## References

[1] Dy GW, Gore JL, Forouzanfar MH, Naghavi M, Fitzmaurice C (2017). Global burden of urologic cancers, 1990-2013. Eur Urol.

[2] Grayson M (2017). Bladder cancer. Nature.

[3] Berdik C (2017). Bladder cancer: 4 big questions. Nature.

[4] Moss TJ, Qi Y, Xi L, Peng B, Kim TB, Ezzedine NE, Mosqueda ME, Guo CC, Czerniak BA, Ittmann M (2017). Comprehensive genomic characterization of upper tract Urothelial carcinoma. Eur Urol.

[5] Humphrey PA, Moch H, Cubilla AL, Ulbright TM, Reuter VE (2016). The 2016 WHO classification of Tumours of the urinary system and male genital organs-part B: prostate and bladder Tumours. Eur Urol.

[6] Alfred Witjes J, Lebret T, Comperat EM, Cowan NC, De Santis M, Bruins HM, Hernandez V, Espinos EL, Dunn J, Rouanne M (2017). Updated 2016 EAU guidelines on muscle-invasive and metastatic bladder Cancer. Eur Urol.

[7] Babjuk M (2017). Trends in bladder Cancer incidence and mortality: success or disappointment?. Eur Urol.

[8] Abufaraj M, Dalbagni G, Daneshmand S, Horenblas S, Kamat AM, Kanzaki R, Zlotta AR, Shariat SF. The role of surgery in metastatic bladder Cancer: a systematic review. Eur Urol. 2017. 10.1016/j.eururo.2017.09.030 PMC8177016 29122377

[9] Babjuk M. Bladder Cancer in the elderly. Eur Urol. 2017. 10.1016/j.eururo.2017.04.018 28478044

[10] Maia MC, Grivas P, Agarwal N, Pal SK. Circulating tumor DNA in bladder Cancer: novel applications and future directions. Eur Urol. 2017. 10.1016/j.eururo.2017.10.011 29097099

[11] Antoni S, Ferlay J, Soerjomataram I, Znaor A, Jemal A, Bray F (2017). Bladder Cancer incidence and mortality: a global overview and recent trends. Eur Urol.

[12] Roupret M (2016). Words of Wisdom. Re: Finasteride Reduces the Risk of Bladder Cancer in a Large Prospective Screening Study. Eur Urol.

[13] Shi X, Sun M, Liu H, Yao Y, Song Y (2013). Long non-coding RNAs: a new frontier in the study of human diseases. Cancer Lett.

[14] Cheng W, Zhang Z, Wang J (2013). Long noncoding RNAs: new players in prostate cancer. Cancer Lett.

[15] Fang XY, Pan HF, Leng RX, Ye DQ (2015). Long noncoding RNAs: novel insights into gastric cancer. Cancer Lett.

[16] Tang Y, Cheung BB, Atmadibrata B, Marshall GM, Dinger ME, Liu PY, Liu T (2017). The regulatory role of long noncoding RNAs in cancer. Cancer Lett.

[17] Zheng R, Du M, Wang X, Xu W, Liang J, Wang W, Lv Q, Qin C, Chu H, Wang M (2018). Exosome-transmitted long non-coding RNA PTENP1 suppresses bladder cancer progression. Mol Cancer.

[18] Zhan Y, Du L, Wang L, Jiang X, Zhang S, Li J, Yan K, Duan W, Zhao Y, Wang L (2018). Expression signatures of exosomal long non-coding RNAs in urine serve as novel non-invasive biomarkers for diagnosis and recurrence prediction of bladder cancer. Mol Cancer.

[19] Heubach J, Monsior J, Deenen R, Niegisch G, Szarvas T, Niedworok C, Schulz WA, Hoffmann MJ (2015). The long noncoding RNA HOTAIR has tissue and cell type-dependent effects on HOX gene expression and phenotype of urothelial cancer cells. Mol Cancer.

[20] Yan H, Bu P (2018). Non-coding RNAs in cancer stem cells. Cancer Lett.

[21] Tseng YY, Moriarity BS, Gong W, Akiyama R, Tiwari A, Kawakami H, Ronning P, Reuland B, Guenther K, Beadnell TC (2014). PVT1 dependence in cancer with MYC copy-number increase. Nature.

[22] Zhan Y, Lin J, Liu Y, Chen M, Chen X, Zhuang C, Liu L, Xu W, Chen Z, He A (2016). Up-regulation of long non-coding RNA PANDAR is associated with poor prognosis and promotes tumorigenesis in bladder cancer. J Exp Clin Cancer Res.

[23] Xie H, Liao X, Chen Z, Fang Y, He A, Zhong Y, Gao Q, Xiao H, Li J, Huang W, Liu Y (2017). LncRNA MALAT1 inhibits apoptosis and promotes invasion by antagonizing miR-125b in bladder Cancer cells. J Cancer.

[24] Chen X, Xie R, Gu P, Huang M, Han J, Dong W, Xie W, Wang B, He W, Zhong G (2019). Long Noncoding RNA Inhibits Self-Renewal and Chemoresistance of Bladder Cancer Stem Cells through Epigenetic Silencing of SOX2. Clin Cancer Res.

[25] He W, Zhong G, Jiang N, Wang B, Fan X, Chen C, Chen X, Huang J, Lin T (2018). Long noncoding RNA BLACAT2 promotes bladder cancer-associated lymphangiogenesis and lymphatic metastasis. J Clin Invest.

[26] Andrew T, Maniatis N, Carbonaro F, Liew SH, Lau W, Spector TD, Hammond CJ (2008). Identification and replication of three novel myopia common susceptibility gene loci on chromosome 3q26 using linkage and linkage disequilibrium mapping. PLoS Genet.

[27] Li Z, Jiang P, Li J, Peng M, Zhao X, Zhang X, Chen K, Zhang Y, Liu H, Gan L, et al. Tumor-derived exosomal lnc-Sox2ot promotes EMT and stemness by acting as a ceRNA in pancreatic ductal adenocarcinoma. Oncogene. 2018. 10.1038/s41388-018-0237-9 29643475

[28] Zhang JJ, Zhu Y, Zhang XF, Liu DF, Wang Y, Yang C, Shi GD, Peng YP, Zhang K, Tian L (2017). Yin Yang-1 suppresses pancreatic ductal adenocarcinoma cell proliferation and tumor growth by regulating SOX2OT-SOX2 axis. Cancer Lett.

[29] Wang P, Xue Y, Han Y, Lin L, Wu C, Xu S, Jiang Z, Xu J, Liu Q, Cao X (2014). The STAT3-binding long noncoding RNA lnc-DC controls human dendritic cell differentiation. Science.

[30] Tsai MC, Manor O, Wan Y, Mosammaparast N, Wang JK, Lan F, Shi Y, Segal E, Chang HY (2010). Long noncoding RNA as modular scaffold of histone modification complexes. Science.

[31] Shafiee M, Aleyasin SA, Vasei M, Semnani SS, Mowla SJ (2016). Down-regulatory effects of miR-211 on long non-coding RNA SOX2OT and SOX2 genes in esophageal squamous cell carcinoma. Cell J.

[32] Hou Z, Zhao W, Zhou J, Shen L, Zhan P, Xu C, Chang C, Bi H, Zou J, Yao X (2014). A long noncoding RNA Sox2ot regulates lung cancer cell proliferation and is a prognostic indicator of poor survival. Int J Biochem Cell Biol.

[33] Su R, Cao S, Ma J, Liu Y, Liu X, Zheng J, Chen J, Liu L, Cai H, Li Z (2017). Knockdown of SOX2OT inhibits the malignant biological behaviors of glioblastoma stem cells via up-regulating the expression of miR-194-5p and miR-122. Mol Cancer.

[34] Harris H (2013). History: non-coding RNA foreseen 48 years ago. Nature.

[35] Joung J, Engreitz JM, Konermann S, Abudayyeh OO, Verdine VK, Aguet F, Gootenberg JS, Sanjana NE, Wright JB, Fulco CP (2017). Genome-scale activation screen identifies a lncRNA locus regulating a gene neighbourhood. Nature.

[36] Lee DF, Su J, Kim HS, Chang B, Papatsenko D, Zhao R, Yuan Y, Gingold J, Xia W, Darr H (2015). Modeling familial cancer with induced pluripotent stem cells. Cell.

[37] Luo M, Li Z, Wang W, Zeng Y, Liu Z, Qiu J (2013). Long non-coding RNA H19 increases bladder cancer metastasis by associating with EZH2 and inhibiting E-cadherin expression. Cancer Lett.

[38] Wu Y, Hu L, Liang Y, Li J, Wang K, Chen X, Meng H, Guan X, Yang K, Bai Y (2017). Up-regulation of lncRNA CASC9 promotes esophageal squamous cell carcinoma growth by negatively regulating PDCD4 expression through EZH2. Mol Cancer.

[39] Li JK, Chen C, Liu JY, Shi JZ, Liu SP, Liu B, Wu DS, Fang ZY, Bao Y, Jiang MM (2017). Long noncoding RNA MRCCAT1 promotes metastasis of clear cell renal cell carcinoma via inhibiting NPR3 and activating p38-MAPK signaling. Mol Cancer.

[40] Chen C, He W, Huang J, Wang B, Li H, Cai Q, Su F, Bi J, Liu H, Zhang B (2018). LNMAT1 promotes lymphatic metastasis of bladder cancer via CCL2 dependent macrophage recruitment. Nat Commun.

[41] Wang Y, Zeng X, Wang N, Zhao W, Zhang X, Teng S, Zhang Y, Lu Z (2018). Long noncoding RNA DANCR, working as a competitive endogenous RNA, promotes ROCK1-mediated proliferation and metastasis via decoying of miR-335-5p and miR-1972 in osteosarcoma. Mol Cancer.

[42] Messemaker TC, van Leeuwen SM, van den Berg PR, t Jong AEJ, Palstra RJ, Hoeben RC, Semrau S, Mikkers HMM (2018). Allele-specific repression of Sox2 through the long non-coding RNA Sox2ot. Sci Rep.

[43] Shahryari A, Rafiee MR, Fouani Y, Oliae NA, Samaei NM, Shafiee M, Semnani S, Vasei M, Mowla SJ (2014). Two novel splice variants of SOX2OT, SOX2OT-S1, and SOX2OT-S2 are coupregulated with SOX2 and OCT4 in esophageal squamous cell carcinoma. Stem Cells.

[44] Zhu F, Qian W, Zhang H, Liang Y, Wu M, Zhang Y, Zhang X, Gao Q, Li Y (2017). SOX2 is a marker for stem-like tumor cells in bladder Cancer. Stem Cell Rep.

