# Correction: Long non-coding RNA SOX2OT promotes the stemness phenotype of bladder cancer cells by modulating SOX2

**DOI:** 10.1186/s12943-023-01822-x

**Published:** 2023-07-18

**Authors:** Yonghao Zhan, Zhicong Chen, Shiming He, Yanqing Gong, Anbang He, Yifan Li, Lianghao Zhang, Xuepei Zhang, Dong Fang, Xuesong Li, Liqun Zhou

**Affiliations:** 1 Department of Urology, The Institute of Urology, Peking University First Hospital, Peking University, National Urological Cancer Center, No. 8 Xishiku street, Beijing, 100034 China; 2 Beijing Key Laboratory of Urogenital Diseases (Male) Molecular Diagnosis and Treatment Center, Beijing, 100034 China; 3 Department of Urology, The First Affiliated Hospital of Zhengzhou University, Zhengzhou, 450003 China

**Correction:** ***Mol Cancer*** **19, 25 (2020)**

10.1186/s12943-020-1143-7

Following publication of the original article [1], the authors identified two errors in Fig. 4k (the Transwell image of sh-NC + Vector SOX2 group was misplaced) and Additional file 2: Figure S2a (the EdU image of sh-NC + Vector NC group was misplaced). The corrected Fig. 4k and Figure S2a using the proper images obtained from the original data can be found below.

**Fig. 4:**
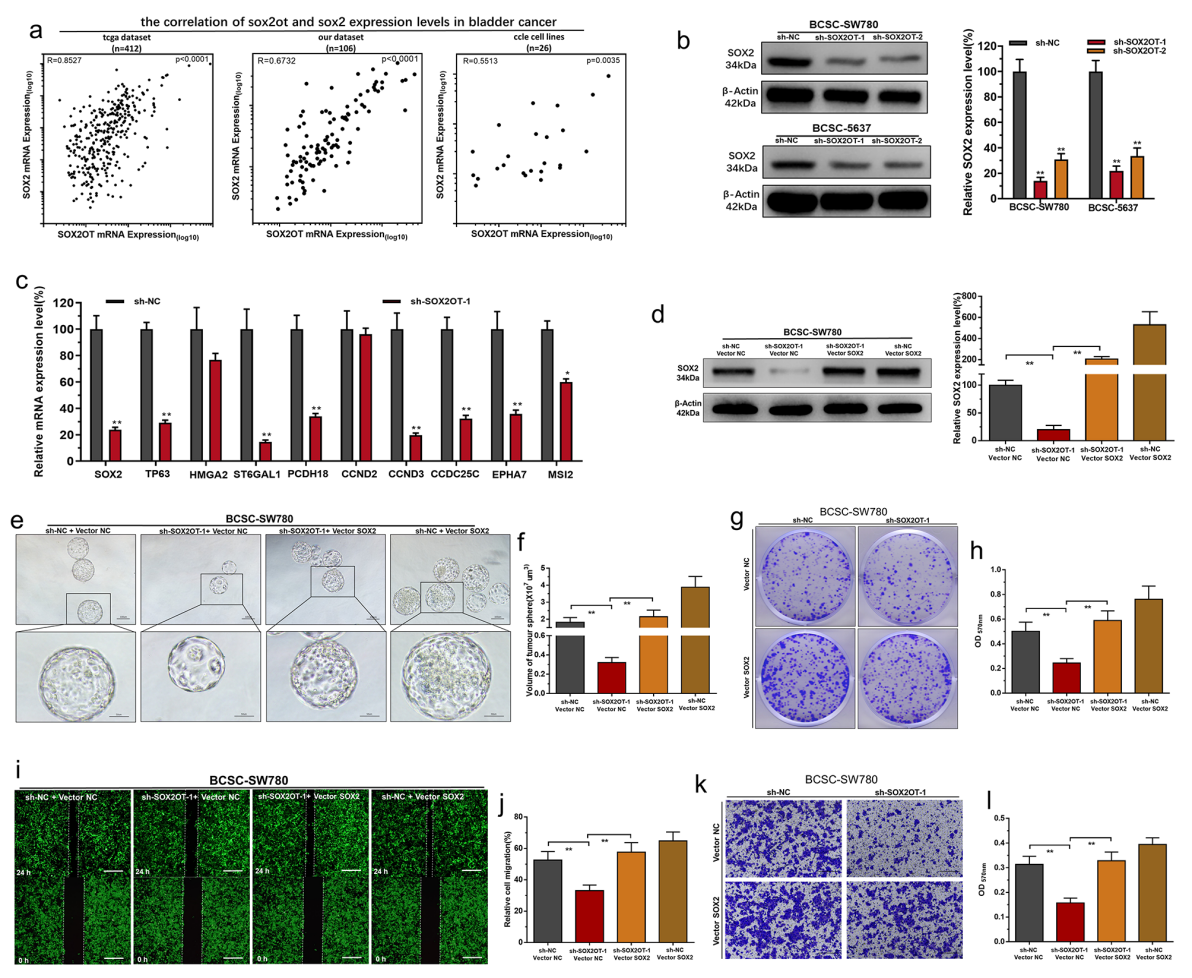
SOX2OT promotes the stemness phenotype of BCSC by modulating SOX2. **a** SOX2OT expression level was positively correlated with SOX2 expression level in BC. **b** Knockdown of SOX2OT decreased SOX2 expression in BCSCs. **c** The expression of SOX2 and SOX2 target genes were determined using RT-qPCR. **d** The SOX2 vector significantly reversed the expression level of SOX2 in BCSCs. **e** and **f** Overexpressing SOX2 significantly reversed the spheroid-formation ability inhibition induced by silencing SOX2OT. **g** and **h** Overexpressing SOX2 significantly reversed the colony forming ability inhibition induced by silencing SOX2OT. **i** and **j** Overexpressing SOX2 significantly reversed the cell migration inhibition induced by silencing SOX2OT. **k** and **l** Overexpressing SOX2 significantly reversed the cell invasion inhibition induced by silencing SOX2OT

## Electronic Supplementary Material

Below is the link to the electronic supplementary material.

**Additional file 2: Figure S2**. Overexpressing SOX2 significantly reversed BCSC proliferation inhibition induced by silencing SOX2OT. a and b: Overexpressing SOX2 significantly reversed BCSC proliferation inhibition induced by silencing SOX2OT.
