# Supplementary figures and images for: Long non-coding RNA SOX2OT promotes the stemness phenotype of bladder cancer cells by modulating SOX2
Source: Mol Cancer. 2020 Feb 4;19:25. doi: 10.1186/s12943-020-1143-7 (PMC6998848; doi:10.1186/s12943-020-1143-7)

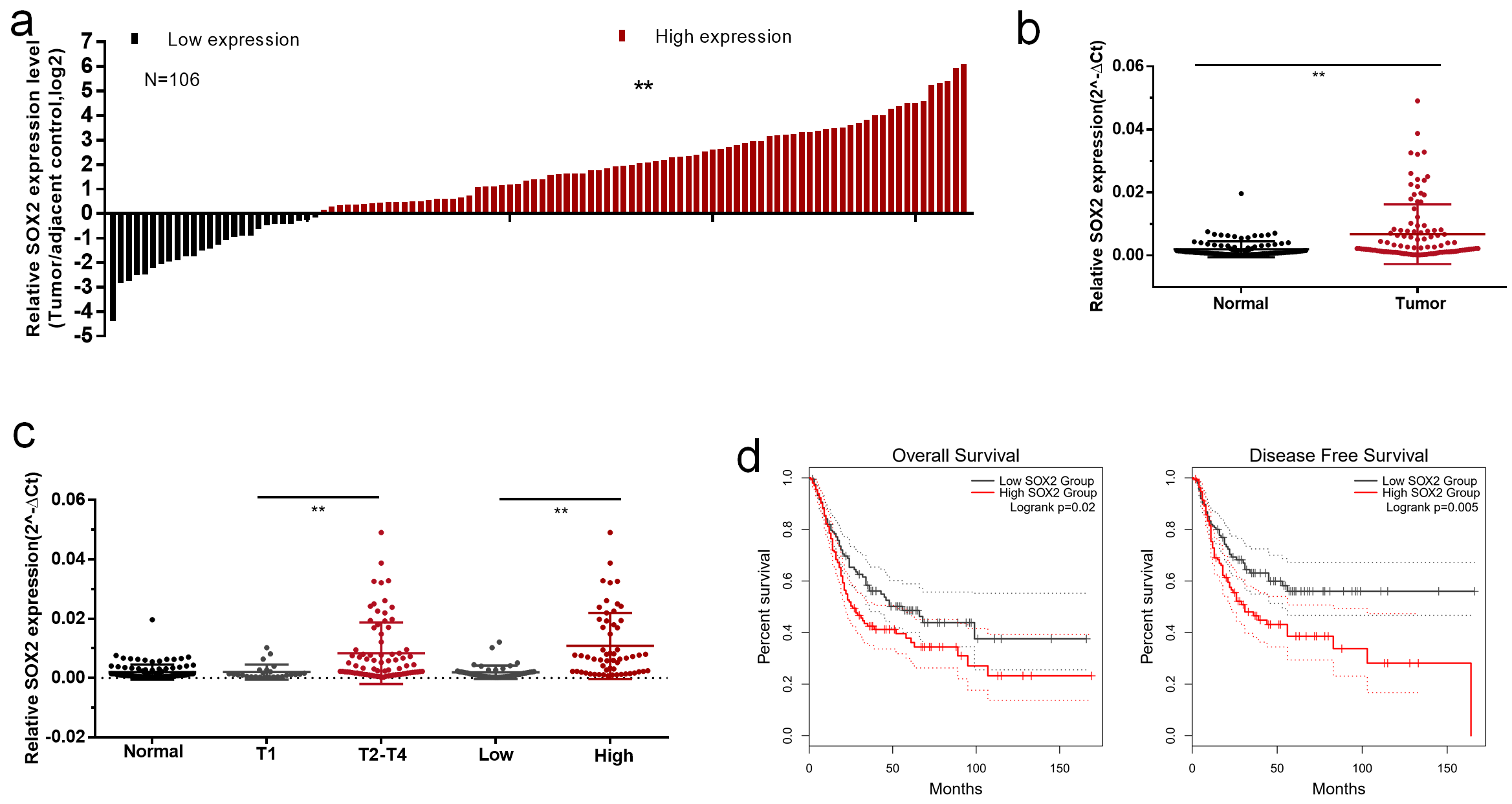

Supplement: Supplementary file 1 — Additional file 1: Figure S1. Expression of SOX2 in bladder cancer. a: The heights of the columns in the chart represent the log2-transformed fold changes (bladder cancer tissue/normal bladder tissue) in SOX2 expression in 106 patients with bladder cancer. b: SOX2 is upregulated in bladder cancer tissues compared with in the corresponding non-tumour tissues. c: SOX2 is upregulated in patients with bladder cancer with an advanced TNM stage and a high histological grade. d: Higher SOX2 expression is related to bladder cancer patients’ shorter overall survival (OS) and disease-free survival (DFS) in TCGA-BLCA. [file 12943_2020_1143_MOESM1_ESM.tif]

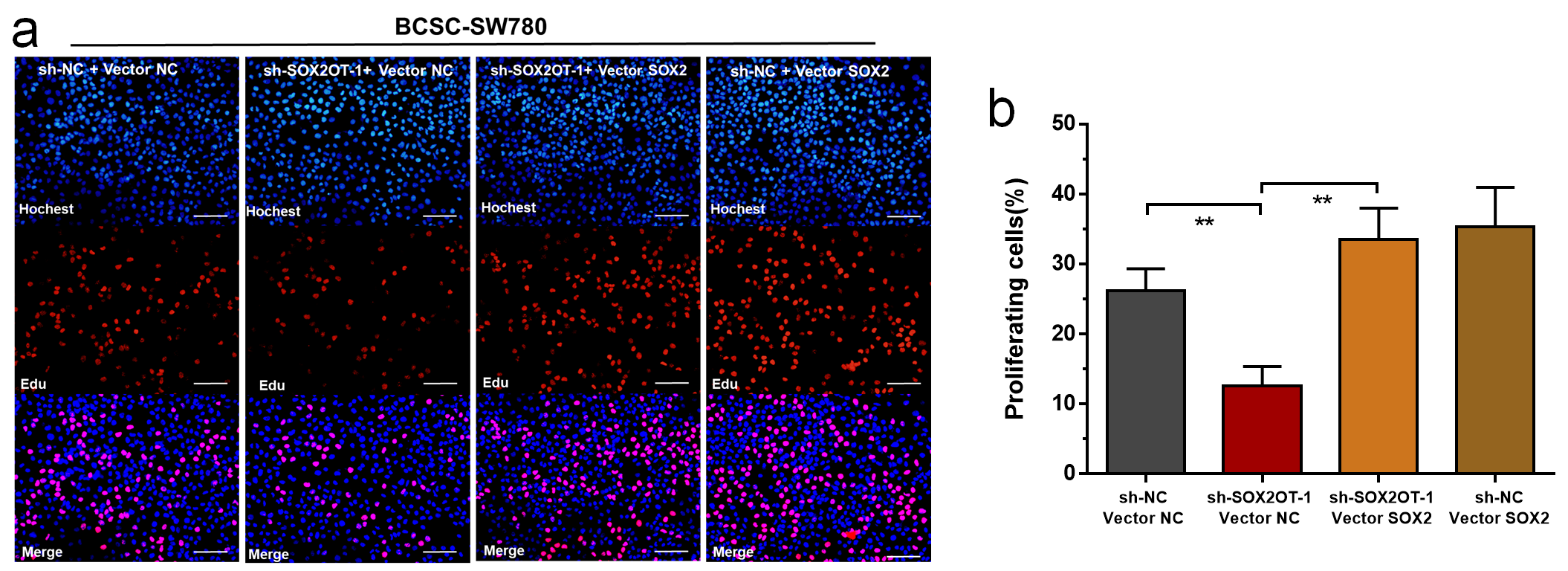

Supplement: Supplementary file 2 — Additional file 2: Figure S2. Overexpressing SOX2 significantly reversed BCSC proliferation inhibition induced by silencing SOX2OT. a and b: Overexpressing SOX2 significantly reversed BCSC proliferation inhibition induced by silencing SOX2OT. [file 12943_2020_1143_MOESM2_ESM.tif]
