# Supplementary material for: Long non-coding RNA SOX2OT promotes the stemness phenotype of bladder cancer cells by modulating SOX2
Source: Mol Cancer. 2020 Feb 4;19:25. doi: 10.1186/s12943-020-1143-7 (PMC6998848; doi:10.1186/s12943-020-1143-7)
Supplement: Supplementary file 4 — Additional file 4: Table S2. The primer sequences included in this study. [file 12943_2020_1143_MOESM4_ESM.docx]

**Table S2. The primer sequences included in this study.**

| **Gene** | **Accession number** | **primer sequences (5’-3’)** | |
| --- | --- | --- | --- |
| SOX2OT | HGNC: 20209 | Forward | TCACTTACAAGACAGCTCTGTTCAGT |
|  |  | Reverse | CAGCATTGCCACGACCTTCCA |
| SOX2 | HGNC: 11195 | Forward | AGGACTGAGAGAAAGAAGAGGAG |
|  |  | Reverse | CGCCGCCGATGATTGTTAT |
| CD44 | HGNC: 1681 | Forward | CTGCCGCTTTGCAGGTGTA |
|  |  | Reverse | CATTGTGGGCAAGGTGCTATT |
| ALDH1 | HGNC: 402 | Forward | CCGTGGCGTACTATGGATGC |
|  |  | Reverse | GCAGCAGACGATCTCTTTCGAT |
| TP63 | HGNC:15979 | Forward | GTCATTTGATTCGAGTAGAGGGG |
|  |  | Reverse | CTGGGGTGGCTCATAAGGT |
| HMGA2 | HGNC: 5009 | Forward | ACCCAGGGGAAGACCCAAA |
|  |  | Reverse | CCTCTTGGCCGTTTTTCTCCA |
| ST6GAL1 | HGNC: 10860 | Forward | ACCCCAATCAGCCCTTTTACA |
|  |  | Reverse | CTGGTCACACAGCGTCATCA |
| CCND2 | HGNC: 1583 | Forward | ACCTTCCGCAGTGCTCCTA |
|  |  | Reverse | CCCAGCCAAGAAACGGTCC |
| CCND3 | HGNC: 1585 | Forward | TACCCGCCATCCATGATCG |
|  |  | Reverse | AGGCAGTCCACTTCAGTGC |
| CDC25C | HGNC: 1727 | Forward | TCTACGGAACTCTTCTCATCCAC |
|  |  | Reverse | TCCAGGAGCAGGTTTAACATTTT |
| PCDH18 | HGNC: 14268 | Forward | GGAACAGAGGGTTGGATCAGT |
|  |  | Reverse | GGCTCGAAATCGAACAGTAGAA |
| EPHA7 | HGNC: 3390 | Forward | AGAACTATACCCCGATACGAACA |
|  |  | Reverse | TGGAAATCCAGTTAGTCCGCA |
| MSI2 | HGNC:18585 | Forward | ACCTCACCAGATAGCCTTAGAG |
|  |  | Reverse | AGCGTTTCGTAGTGGGATCTC |
| E-cadherin | HGNC: 1748 | Forward | ATTTTTCCCTCGACACCCGAT |
|  |  | Reverse | TCCCAGGCGTAGACCAAGA |
| N-cadherin | HGNC: 1759 | Forward | AGCCAACCTTAACTGAGGAGT |
|  |  | Reverse | GGCAAGTTGATTGGAGGGATG |
| Vimentin | HGNC: 12692 | Forward | AGTCCACTGAGTACCGGAGAC |
|  |  | Reverse | CATTTCACGCATCTGGCGTTC |
| β-actin | HGNC: 132 | Forward | GCGGACTATGACTTAGTTGCGTTACA |
|  |  | Reverse | TGCTGTCACCTTCACCGTTCCA |
| miR-200a | MIMAT0000682 | Forward | GGGCTAACACTGTCTGGTAAC |
| miR-200b | MIMAT0000318 | Forward | CGGTAATACTGCCTGGTAATGATG |
| miR-200c | MIMAT0000617 | Forward | TAATACTGCCGGGTAATGATGGA |
| miR-429 | MIMAT0001536 | Forward | CGGGCTAATACTGTCTGGTAAAAC |
| miR-141 | MIMAT0000432 | Forward | GGGCTAACACTGTCTGGTAAAGA |
| U6 |  | Forward | CTCGCTTCGGCAGCACA |
|  |  | Reverse | AACGCTTCACGAATTTGCGT |
