# Supplementary material for: Long non-coding RNA SOX2OT promotes the stemness phenotype of bladder cancer cells by modulating SOX2
Source: Mol Cancer. 2020 Feb 4;19:25. doi: 10.1186/s12943-020-1143-7 (PMC6998848; doi:10.1186/s12943-020-1143-7)
Supplement: Supplementary file 5 — Additional file 5: Table S3. Results of Bioinformation analysis. [file 12943_2020_1143_MOESM5_ESM.docx]

**Table S3. Results of Bioinformation analysis.**

| microRNAs | lncRNAS | mRNA |
| --- | --- | --- |
| hsa-miR-15a-3p | SOX2OT | SOX2 |
| hsa-miR-19a-5p | SOX2OT | SOX2 |
| hsa-miR-19b-3p | SOX2OT | SOX2 |
| hsa-miR-26a | SOX2OT | SOX2 |
| hsa-miR-139-5p | SOX2OT | SOX2 |
| hsa-miR-141-5p | SOX2OT | SOX2 |
| hsa-miR-200a | SOX2OT | SOX2 |
| hsa-miR-200b | SOX2OT | SOX2 |
| hsa-miR-200c | SOX2OT | SOX2 |
| hsa-miR-205-5p | SOX2OT | SOX2 |
| hsa-miR-340 | SOX2OT | SOX2 |
| hsa-miR-365 | SOX2OT | SOX2 |
| hsa-miR-429 | SOX2OT | SOX2 |
| hsa-miR-490-3p | SOX2OT | SOX2 |
| hsa-miR-539 | SOX2OT | SOX2 |
| hsa-miR-543 | SOX2OT | SOX2 |
| hsa-miR-574-5p | SOX2OT | SOX2 |
| hsa-miR-762 | SOX2OT | SOX2 |
| hsa-miR-876-5p | SOX2OT | SOX2 |
| hsa-miR-942 | SOX2OT | SOX2 |
| hsa-miR-1226 | SOX2OT | SOX2 |
| hsa-miR-1236 | SOX2OT | SOX2 |
| hsa-miR-1273 | SOX2OT | SOX2 |
| hsa-miR-3176 | SOX2OT | SOX2 |
| hsa-miR-3678 | SOX2OT | SOX2 |
| hsa-miR-3679-3p | SOX2OT | SOX2 |
| hsa-miR-3922 | SOX2OT | SOX2 |
| hsa-miR-4731-5p | SOX2OT | SOX2 |
| hsa-miR-5193 | SOX2OT | SOX2 |
