# Supplementary figures and images for: Correction: Long non-coding RNA SOX2OT promotes the stemness phenotype of bladder cancer cells by modulating SOX2
Source: Mol Cancer. 2023 Jul 18;22:115. doi: 10.1186/s12943-023-01822-x (PMC10353153; doi:10.1186/s12943-023-01822-x)

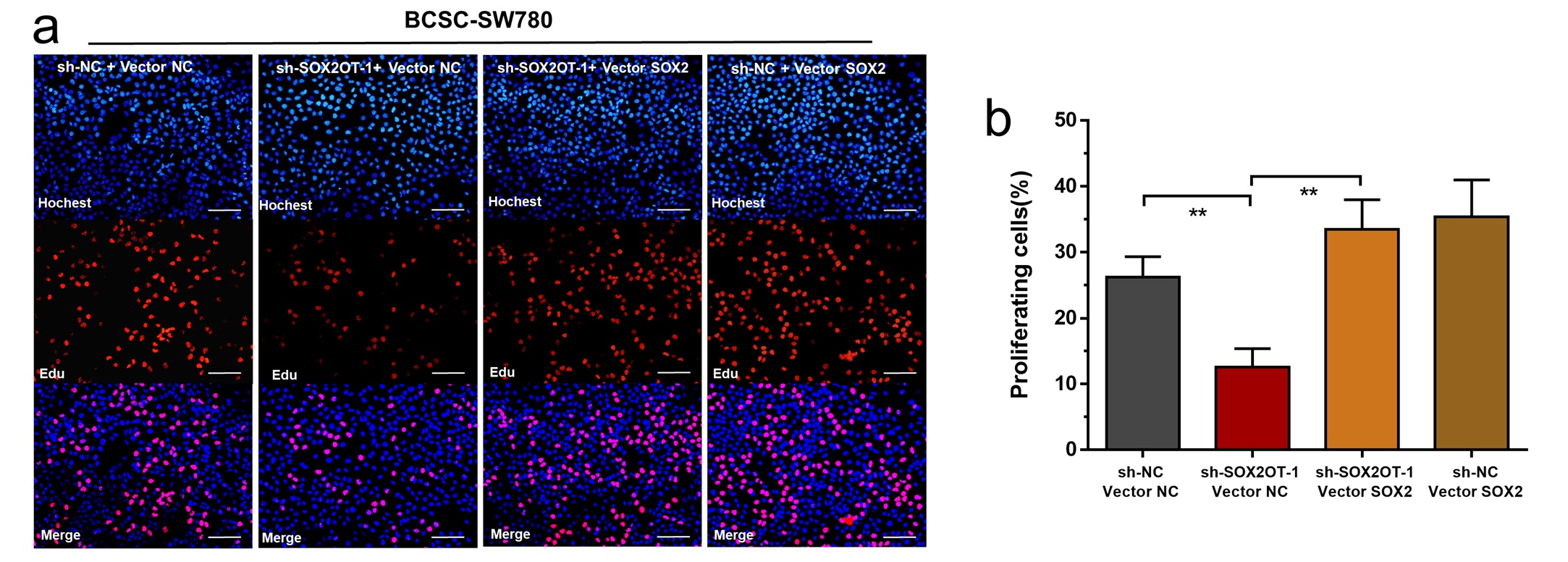

Supplement: Supplementary file 1 — Additional file 2: Figure S2. Overexpressing SOX2 significantly reversed BCSC proliferation inhibition induced by silencing SOX2OT. a and b: Overexpressing SOX2 significantly reversed BCSC proliferation inhibition induced by silencing SOX2OT. [file 12943_2023_1822_MOESM2_ESM.jpg]
